# Spatial variation and predictors of incomplete pneumococcal conjugate vaccine (PCV) uptake among children aged 12–35 months in Ethiopia: spatial and multilevel analyses

**DOI:** 10.3389/fpubh.2024.1344089

**Published:** 2024-05-28

**Authors:** Aklilu Habte Hailegebireal, Samuel Hailegebreal, Lire Lemma Tirore, Biruk Bogale Wolde

**Affiliations:** ^1^School of Public Health, College of Medicine and Health Sciences, Wachemo University, Hosaina, Ethiopia; ^2^Department of Health Informatics, College of Medicine and Health Sciences, School of Public Health, Wachemo University, Hosaina, Ethiopia; ^3^Menzies School of Health Research, Charles Darwin University, Darwin, NT, Australia; ^4^School of Public Health, College of Medicine and Health Sciences, Wachemo University, Hossana, Ethiopia; ^5^School of Public Health, College of Medicine and Health Sciences, Mizan Tepi University, Mizan Aman, Ethiopia

**Keywords:** incomplete immunization, children, PCV, predictors, spatial, Ethiopia

## Abstract

**Background:**

Despite the Ethiopian government included the Pneumococcal Conjugate Vaccine (PCV) in the national expanded program for immunization in 2011, only 56% of children aged 12–23 months received the full dose of PCV. Despite some studies on PCV uptake in Ethiopia, there was a dearth of information on the geographical distribution and multilevel factors of incomplete PCV uptake. Hence, this study aimed to identify the spatial variations and predictors of incomplete PCV uptake among children aged 12–35 months in Ethiopia.

**Methods:**

The study was based on an in-depth analysis of 2016 Ethiopia Demographic Health Survey data, using a weighted sample of 3,340 women having children aged 12–35 months. Arc-GIS version 10.7 and SaTScan version 9.6 statistical software were used for the spatial analysis. To explore spatial variation and locate spatial clusters of incomplete PCV, the Global Moran's I statistic and Bernoulli-based spatial scan (SaTScan) analysis were carried out, respectively. A multilevel mixed-effect multivariable logistic regression was done by STATA version 16. Adjusted odds ratio (AOR) with its corresponding 95% CI was used as a measure of association, and variables with a *p* < 0.05 were deemed as significant determinants of incomplete PCV.

**Results:**

The overall prevalence of incomplete PCV in Ethiopia was found to be 54.0% (95% CI: 52.31, 55.69), with significant spatial variation across regions (Moran's I = 0.509, *p* < 0.001) and nine most likely significant SaTScan clusters. The vast majority of Somali, southeast Afar, and eastern Gambela regions were statistically significant hot spots for incomplete PCV. Lacking ANC visits (AOR = 2.76, 95% CI: 1.91, 4.00), not getting pre-birth Tetanus injections (AOR = 1.84, 95% CI: 1.29, 2.74), home birth (AOR = 1.72, 95% CI: 1.23, 2.34), not having a mobile phone (AOR = 1.64, 95% CI: 1.38, 1.93), and residing in a peripheral region (AOR = 4.63; 95% CI: 2.34, 9.15) were identified as statistically significant predictors of incomplete PCV.

**Conclusion:**

The level of incomplete PCV uptake was found to be high in Ethiopia with a significant spatial variation across regions. Hence, the federal and regional governments should collaborate with NGOs to improve vaccination coverage and design strategies to trace those children with incomplete PCV in peripheral regions. Policymakers and maternal and child health program planners should work together to boost access to maternal health services like antenatal care and skilled delivery services to increase immunization coverage.

## Background

Pneumococcal illness is an acute infection caused by the Streptococcus pneumoniae bacteria, which can cause serious invasive diseases such as pneumonia, meningitis, and septicemia as well as milder but more common illnesses such as sinusitis and otitis media (ear infections) in children (1). In 2015, these infections were responsible for 294,000 of the 5.83 million global deaths among children under the age of five (2). Streptococcus pneumoniae, the causal agent, frequently colonizes the human nasopharynx and is primarily transmitted through respiratory droplets (3, 4). Infants and young children are its main reservoirs, with greater carriage rates among children in low- and middle-income countries (LMICs) (3).

Pneumonia is a type of acute respiratory tract infection that affects the lungs and their parenchyma. It is the single most common infectious cause of death among children globally, killing 740, 180 children under the age of five in 2019, making up 14% of all deaths in children under the age of five but 22% of the deaths in children aged one to five (5). Sub-Saharan Africa carries the greatest (50%) burden of worldwide under-five mortality from pneumonia (6). A considerable number of children in Ethiopia had not been immunized (7). As a result, infant mortality in Ethiopia is among the highest in the world, and many of these deaths were thought to be caused by vaccine-preventable diseases (8). Pneumonia is the top cause of death among children under the age of five in Ethiopia. It is estimated that 3,370,000 children are affected by pneumonia each year, accounting for 20% of all causes of death and killing over 40,000 children under the age of five (9).

Vaccines are considered one of the most safe and cost-efficient interventions to combat childhood illness and death (10). Vaccination currently averts 2–3 million deaths worldwide each year (11). Pneumococcal conjugate vaccine (PCV) is the most effective intervention to prevent pneumonia in children (3, 12, 13). A double-blind, randomized controlled experiment showed that 13-valent PCV provided 78% protection against pneumococcus serotype 6B (BHN418) acquisition (14). Even though various vaccines have been manufactured to minimize or eliminate the burden of infections caused by S. pneumonia, the WHO presently recommends two types of vaccines, namely the unconjugated 23-valent polysaccharide vaccine (PPSV) and the 10- or 13-valent conjugated polysaccharide vaccine (PCV) (3, 15). Based on the national epidemiology of S. pneumoniae serotypes and cost, the WHO recommended that nations utilize either 10- or 13-valent vaccines in their national immunization programs (3). The 10-valent pneumococcal conjugate vaccine (PCV10) is made up of capsular polysaccharides isolated from ten serotypes and is conjugated to either protein D, tetanus toxoid, or diphtheria toxoid (3).

The Global Vaccine Action Plan (GVAP) suggests all countries should plan to reach 90% national coverage of all vaccines at the end of 2020. However, as per the WHO report, worldwide immunization coverage was only 85% in 2017, with 19.9 million children missing out on basic life-saving vaccines (11). By the end of 2022, PCV had been adopted in 155 WHO Member States, and global third dose (PCV3) coverage was predicted to be 60%. There is significant regional variation, with the WHO European Region expected to have 83% coverage, whereas the WHO Western Pacific Region has only 23% (11).

The Ethiopian government included the PCV10 in the national infant immunization program in November 2011 (16). It was given to children in three doses, during the sixth, tenth, and fourteenth weeks of life. However, when it was first introduced in Ethiopia, all children under the age of one had to be vaccinated as part of a catch-up program (16–18). However, the 2016 Ethiopian Demographic Health Survey (2016 EDHS) report showed that only 56% of children aged 12–23 months received the full dose of PCV (19). This result lags far behind the EPI and the GVAP, which set national vaccine coverage goals of 90% by 2020 (11). According to the reports, there is a significant disparity in immunization coverage between regions due to different challenges and possibilities. Access to health services, family economic status, place of delivery, antenatal care (ANC) visits, maternal education, knowledge of immunization, access to mass media, place of residence, and religion were also identified as factors influencing childhood vaccination (7, 20–22).

Although numerous studies on vaccine uptake have been undertaken in Ethiopia, they have primarily focused on overall immunization, with little emphasis placed on the spatial distribution and multilevel determinants of incomplete PCV uptake. Hence, this study aimed to identify the spatial variations as well as the individual and community-level predictors of missing PCV among children aged 12–35 months in Ethiopia. By incorporating spatial analysis into the assessment of PCV coverage, policymakers can make data-driven decisions to enhance vaccine delivery mechanisms, address disparities in coverage, and ultimately improve vaccination rates nationwide. This approach not only supports the achievement of vaccination targets but also contributes to the overarching goal of reducing pneumococcal-related morbidity and mortality, thereby advancing public health outcomes in Ethiopia.

## Methods and materials

### Study setting and data source

Ethiopia is the second most populous country in Africa, located in the Horn of Africa, at 3^0^-15^0^ N latitude and 33^0^-48^0^ E longitude. The country is administratively segmented into 9 geographical regions (Tigray, Afar, Amhara, Oromiya, Somali, Benishangul Gumuz, Southern Nations Nationalities and Peoples (SNNP), Gambela, and Harari) and two administrative cities (Addis Ababa and Dire Dawa)(19). This study was based on a secondary analysis of the women's (IR) file of 2016 EDHS, which was carried out between January 18, to June 27, 2016.

### Study population and eligibility criteria

The study population consisted of women having children aged 12–35 months who resided in the selected clusters and had complete information on vaccination status for PCV. Women whose geographical locations were not available through the global positioning system (GPS) were excluded from the spatial analysis.

### Sampling procedure and data collection tools

To select study participants, the 2016 EDHS used a stratified, two-stage cluster sampling approach, with each region divided into urban and rural areas. First, 645 enumeration areas (EAs) (202 and 443 from urban and rural areas, respectively) were selected using a probability proportional to EA size. Then, in each of the selected EAs, a household listing operation was run from September to December 2015, and the resulting lists were used as a sampling frame for the selection of households. The newly formed household listing was then used to select a fixed number of 28 households per cluster with an equal probability of systematic selection. Finally, a total of 3,340 women with children aged 12–35 months were included in the analysis. The detailed methodology is available in the 2016 EDHS final report (19). Data were collected by using the Woman's Questionnaire, which contains background characteristics, birth history, maternal health service characteristics, vaccination status, and childhood illnesses.

### Measurement of variables of the study

#### Outcome variable

The outcome variable was having incomplete PCV schedules among children aged 12–35 months. The status of each dose of PCV was assessed, and if a child received all three doses of PCV (PCV1= h54_1, PCV2= h55_1, and PCV3 = h56_1), it was classified as ‘complete=0”, otherwise considered as “incomplete=1”. Written vaccination records (including the infant's immunization card and other health cards) and verbal reports from mothers were used to assess the vaccination status.

#### Explanatory variables

Following a review of relevant and related studies, individual and community-level determinants of incomplete PCV uptake were identified (Table 1).

**Table 1:** List of potential predictors of incomplete PCV uptake extracted from the EDHS 2016 report.

**Individual-level factors (maternal and child characteristics)**	
**Variables**	**Description**
Age of mother	The respondent's age, expressed in years, at the time of the survey and categorized as 15–19, 20–34, and 35–49 years
Marital status	Categorized as in marital relationships or not in a marital relationship
Level of education	Categorized as no education, primary and secondary/higher education
Family size	Number of household members at the time of data collection and categorized as ≤ 5 or >5
Age of child	The age of children expressed in months at the time of the survey and categorized as 12–23 and 24–35 months
Sex of head of household	Percent distribution of households by sex of head of household as male and female
Wealth index	Calculated using straightforward information on a household's ownership of certain goods, such as televisions and bicycles; housing materials; livestock, crop production, and access to water, sanitation, and hygiene. Finally, it was grouped into richest, richer, middle, poorer, and poorest.
Parity	The number of living children the woman had at the time of the survey and grouped into Primiparous, Multiparous, and Grand multiparous
Antenatal care	Number of women who received antenatal care for their last birth, which was initially reported in continuous form and then grouped as no antenatal care, 1 visit, 2–3 visits, 4+ visits
Pregnancy status during last childbirth	Categorized unwanted (wanted later and not wanted at all) and wanted (wanted then)
Place of delivery	Percent distribution of live births in the past 5 years by place of delivery as of health facility and home.
Postnatal checkup	The proportion of women giving birth within 2 years preceding the survey, who received a postnatal check for the most recent live birth within the first 2 months following childbirth grouped as yes or no
Tetanus toxoid injection	Percentage of women with a live birth in the 5 years preceding the survey receiving two or more injections prior to the most recent live birth
Media exposure	The number of women exposed to specific media at various frequencies, such as reading a newspaper, watching television, and listening to the radio. Categorized as: not at all, less than once a week, and at least once a week
Difficulty in accessing healthcare	The ease of accessing health care for themselves when they were sick was grouped as “Not a big problem” or “Big problem”
Difficulty in accessing money	The ease of getting money needed for treatment when they were sick which was grouped as “Not a big problem” or “A big problem”
Distance to a health facility	The ease of distance to access treatment when they were sick which was grouped as “Not a big problem” or “A big problem”
Autonomy in decision-making	Was assessed by using three questions about who makes the final decision for the family on large property purchases, visits to relatives, and health care. The response categories were (i) woman alone, (ii) woman and husband/partner, (iii) husband/partner alone, (iv) someone else, and (v) others. For each question, responses (i) or (ii) got a score of 1, indicating good decision-making capacity, whereas the remaining responses received a score of 0, indicating weak decision-making capacity. Each of the three components' responses were summed together to yield an overall score ranging from 0 to 3. Finally, a composite score was divided into two distinct groups: low and high for “0 to 2” and “3” scores (23)
**Community level factors**	
Residence	The place of residence of respondents at the time the survey was carried out.
Region	The geographical area in which the woman lived at the time of the survey. Small peripheral regions (Afar, Somalia, Benishangul, and Gambella), Major central regions (Tigray, Amhara, and Oromia), and Metropolitans (Addis Ababa, Dire Dawa, and the Harari region) have been created (24, 25).

### Data management and statistical analysis

Using STATA version 16, the data was extracted, recoded, and cleaned. In addition, ArcGIS version 10.7, and SaTScan version 9.6 were used for spatial analysis. A weighting was done to account for the unequal likelihood of selection among strata caused by the non-proportional distribution of samples to different areas. This helps to restore the statistical representativeness of the survey, which is essential for reliable estimation (26). Descriptive statistics such as frequency and percentage of different variables were estimated and displayed using texts, and tables.

### Spatial analysis

The weighted proportions of the overall PCV uptake (Complete vs. Incomplete/missed) were computed in Microsoft Excel 2016 (CSV format) and then imported into ArcGIS 10.7. The CSV file was then combined with the geographic coordinates located in the shape file using the unique identification code of each cluster.

### Spatial autocorrelation

Global spatial autocorrelation was carried out using the Global Moran's I statistic (Moran's I) to determine whether the overall pattern of incomplete PCV uptake is clustered, dispersed, or random throughout the study areas. A Moran's I value close to 1 indicates a significant positive autocorrelation (the outcome of interest is clustered/non-random) and close to −1 indicates negative spatial autocorrelation (the outcome is dispersed) across enumeration areas (EAs). Moran's I value near zero implies that the spatial distribution was random (27, 28). In the current analysis, a statistically significant Moran's I (*p* < 0.001) leads to the rejection of the null hypothesis (the outcome is randomly distributed) and indicates the presence of spatial autocorrelation (28).

### Hot spot analysis (Getis-Ord Gi^*^ statistics)

Getis-Ord Gi^*^ statistics were calculated for each location to detect significant hot and cold spots for children with incomplete PCV. To determine the statistical significance of clustering, the Z-score was obtained, and the *p*-value was determined. Statistical output with a high GI suggests a “hot spot,” whereas one with a low GI indicates a “cold spot” (29, 30).

### Spatial interpolation

Spatial interpolation was done by using the Ordinary Kriging technique to forecast the status of incomplete/missed PCV in unsampled areas by using evidence from the sampled EAs. The ordinary Kriging spatial interpolation approach was chosen from among several interpolation techniques because it has a low residual and mean square error (31, 32).

### Spatial scan statistical analysis

Using Kuldorff's SaTScan version 9.6 statistical software, the Bernoulli-based model was employed to detect the statistically significant spatial clusters with incomplete PCV uptake. A Bernoulli-based model was used in which events at particular places were analyzed, whether children were fully vaccinated (Control) or not (Case). Scan statistics scanned the space gradually to determine the number of observed and expected observations inside the window at each cluster. The scanning window with the maximum likelihood was the most likely and high-performing cluster for being a case, and the level of significance was determined at a *p*-value < 0.05.

### Multilevel mixed-effect logistic regression

To account for the hierarchical nature of EDHS data, a multilevel multivariable logistic regression analysis was carried out to identify community and individual-level predictors of incomplete PCV uptake. Running a multilevel analysis upon such hierarchical data allows for minimal biased parameter estimates (33, 34).

### Model building and selection

#### Fixed effects (measures of association)

Initially, a multilevel bivariable logistic regression analysis was run to examine the effect of each explanatory variable on the outcome variable, and variables with *p* < 0.25 were included in the multilevel multivariable logistic regression. The Variance Inflation Factor (VIF) was used to detect multicollinearity among covariates at a cutoff point of 10 and found none (the VIF ranged from 1.01 to 2.27 with a mean of 1.54). Finally, a multilevel multivariable logistic regression analysis was performed to find significant predictors of incomplete PCV uptake, and statistically significant variables at *p* < 0.05 were reported along with their 95% confidence intervals.

#### Random effects (measures of variation)

Four distinct models were fitted: the first (model 1) was without any covariate (empty model), the second (model 2) with only individual-level factors, the third (model 3) with merely community-level variables, and the fourth (Model 4/full model) with both individual- and community-level variables. Intra-class correlation coefficient (ICC), median odds ratio (MOR), and proportional change in variance (PCV) metrics were computed for measures of variation.

ICC is a measure of the degree of heterogeneity of not having full PCV between clusters, and it was determined using:

$ICC=\frac{\text{var}\left(\text{b}\right)}{\text{Var}\left(\text{b}\right)+\text{Var}\left(\text{w}\right)}=$ $\frac{\text{var}\left(\text{b}\right)}{\text{Var}\left(\text{b}\right)+3.29}$; where Var(b) is the estimated variance each model and Var(w) is a predicted individual variance component, which is π2/3 ≈3.29.

The PCV was used to evaluate the contribution of individual- and/or community-level factors to the overall variation in the null model, and it was determined by:

$PCV=\frac{\left(\text{Va}-\text{Vb}\right)}{\text{Va}}*100$, where, V_a_ is the variance of the initial model (null model), and V_b_=variance of the subsequent models (models 2, 3, and 4).

MOR measured the unexplained heterogeneity in the odds of incomplete PCV uptake from one cluster to another one and is determined by using:


$$\begin{array}{l} x=\;\frac{\exp(\sqrt{2^{*}\text{Vb}}}{2a}*0.6745\approx \text{exp}(\sqrt{2^{*}\text{V}b}) \end{array}$$

### Model fitness

Deviance = −2 ^*^ Log Likelihood (LL), Schwarz's Bayesian Information Criterion (BIC), and Akaike's Information Criterion (AIC) were used to compare models. Finally, the best-fitted model was determined to be the one with the highest likelihood ratio test and the lowest deviance.

## Results

### Background characteristics of study participants

A weighted sample of 3,340 women having children aged 12–35 months was included in the current study. The mean (±SD) age of women was 28.96 (±6.59) years. On the other hand, the mean (±SD) age of the children was 21.98 (±6.89), with the majority (57.0%) of them falling between the ages of 12–23 months. The vast majority of the respondents (90.9%) were from major central regions. Nearly two-thirds of the mothers (63.9%) and one-fourth (24.7%) had no formal education and were in the poorest wealth quintile, respectively. The highest proportions of children with incomplete PCV uptake were reported among women from peripheral regions (65.0%), and who had no formal education (61.1%) (Table 2).

**Table 2:** The background characteristics of study participants across the uptake of PCV in Ethiopia, EDHS 2016.

**Variable categories**	**Total [weighted frequency (%)]**	**Status of PCV uptake**		**COR (95%CI)**
		**Incomplete [*****n*** **(%)]**	**Fully [*****n*** **(%)]**	
**Maternal age (in years)**				
15–24	784 (23.5)	410 (52.4)	374 (47.6)	Ref.
25–34	1,760 (52.7)	917 (52.1)	843 (47.9)	1.04 (0.72, 1.48)
35–49	795 (23.8)	476 (59.8)	319 (40.2)	1.38 (0.94, 2.03)
Child's age (in months)				
12–23	1,905 (57.0)	963 (50.6)	942 (49.4)	Ref.
24–35	1,435 (43.0)	841 (58.6)	594 (41.4)	1.66 (1.24, 2.22)^*^
Mean (±SD) age	21.98 (± 6.89)			
**Sex of child**				
Male	1,674 ()	896 (53.5)	778 (46.5)	Ref.
Female	1,666	907 (54.5)	758 (45.5)	1.03 (0.78, 1.35)
**Regions**				
Major central regions	3,035 (90.9)	1,661 (54.7)	1,374 (45.3)	9.61 (5.61, 16.4)^*^
Peripheral	197 (5.9)	128 (65.0)	69 (35.0)	25.18 (13.56, 26.73)^*^
Metropolitans	107 (3.2)	14 (13.5)	93 (86.5)	Ref.
**Religion**				
Orthodox	1,237 (37.0)	508 (41.1)	729 (58.9)	Ref.
Muslim	1,222 (36.6)	827 (67.6)	395 (32.4)	4.00 (2.69, 5.93)^*^
Protestant	761 (22.8)	383 (50.3)	378 (49.7)	1.80 (1.21, 2.69)^*^
Catholic	43 (1.3)	22 (51.0)	21 (49.0)	2.78 (0.84, 9.11)
Others	77 (2.3)	64 (83.3)	13 (16.7)	13.10 (3.71, 26.26)^*^
**Marital status**				
In marital relationship	3,093 (93.7)	1,653 (53.5)	1,439 (46.5)	Ref.
Not in a marital relationship	247 (6.3)	150 (60.9)	97 (39.1)	1.29 (0.75, 2.23)
**Residence**				
Urban	391 (11.7)	114 (29.2)	277 (71.8)	Ref.
Rural	2,948 (88.3)	1,689 (57.3)	1,259 (42.7)	5.52 (3.42, 8.90)^*^
**Wealth index combined**				
Poorest	824 (24.7)	539 (65.4)	285 (34.5)	4.23 (2.49, 7.18)^*^
Poorer	731 (21.8)	418 (57.1)	313 (42.9)	2.92 (1.78, 4.76)^*^
Middle	677 (20.3)	393 (58.1)	283 (41.9)	3.14 (1.89, 5.22)^*^
Richer	590 (17.7)	300 (50.9)	290 (49.1)	2.42 (1.50, 3.92)^*^
Richest	518 (15.5)	154 (29.7)	364 (70.3)	Ref.
**Educational status**				
No education	2,133 (63.9)	1,303 (61.1)	830 (38.9)	4.85 (2.51, 9.39)^*^
Primary	938 (28.1)	438 (46.6)	501 (53.4)	2.93 (1.53, 5.61)^*^
Secondary and higher	269 (8.0)	64 (23.6)	205 (74.4)	Ref.

Ref, Reference category;

* significant at p < 0.25.

### Obstetric and health service-related characteristics

The majority (46.3%) of women were multiparous. In terms of maternal health service utilization, 37.4% and 66.7% of respondents, respectively, did not get Antenatal care (ANC) and gave birth at home during their most recent pregnancy. A large proportion of women, 72.7%, 80.9%, and 92.8%, were never exposed to radio, television, and newspapers, respectively. Only 17.5% of women owned a mobile phone and 4.2% were enrolled in health insurance schemes. Distance and money were reported impediments to medical care in 58.7% and 61.3% of women, respectively. The highest proportions of children with incomplete PCV uptake were reported among women who had no ANC visit (72.5%), no pre-birth Tetanus toxoid injection, and who gave birth at home (63.1%) (Table 3).

**Table 3:** Obstetric and health service-related characteristics of study participants across the uptake of PCV in Ethiopia, EDHS 2016.

**Variable categories**	**Total [weighted frequency (%)]**	**Status of PCV uptake**		**COR (95%CI)**
		**Incomplete** **[*****n*** **(%)]**	**Full [*****n*** **(%)]**	
**Parity**				
Primiparous	685 (20.5)	328 (47.9)	357 (52.1)	Ref.
Multiparous	1,546 (46.3)	775 (50.1)	771 (49.9)	1.08 (0.72, 1.61)
Grand multiparous	1,109 (33.2)	701 (63.2)	408 (36.8)	1.58 (1.08, 2.30)^*^
**Frequency of ANC**				
No visit	1,251 (37.4)	907 (72.5)	34,427.5 ()	4.13 (2.90, 5.88)^*^
One visit	137 (4.1)	88 (64.1)	49 (35.9)	3.95 (1.99, 7.82)^*^
2–3 visits	866 (25.9)	417 (48.1)	449 (51.9)	1.55 (1.09, 2.21)^*^
≥4 visits	1,086 (32.5)	393 (36.1)	694 (63.9)	Ref.
**Tetanus vaccine before delivery**				
Yes	1,801 (53.9)	771 (42.8)	1,030 (57.2)	Ref.
No	1,539 (46.1)	1,033 (67.1)	506 (32.9)	2.77 (2.03, 3.78)^*^
**Place of delivery**				
Home	2,229 (66.7)	1,406 (63.1)	822 (36.9)	2.71 (1.93, 3.80)^*^
Health facilities	1,111 (33.3)	397 (35.8)	714 (64.2)	Ref.
Postnatal care service				
Yes	278 (8.3)	94 (33.6)	185 (66.4)	1.89 (1.17, 3.04)^*^
No	3,061 (91.7)	1,710 (55.7)	1,351 (44.3)	Ref.
**Planning status of pregnancy**				
Wanted	2,503 (74.9)	1,378 (55.1)	1,123 (44.9)	Ref.
Unwanted	837 (25.1)	426 (50.9)	411 (49.1)	0.75 (0.69, 1.16)
**Listen to radio**				
Not at all	2,427 (72.7)	1,430 (58.9)	996 (41.1)	2.38 (1.54, 3.67)^*^
Less than once a week	453 (13.6)	191 (42.8)	262 (57.2)	1.15 (0.68, 1.94)
At least once a week	460 (13.8)	182 (39.6)	278 (61.4)	Ref.
**Watching TV**				
Not at all	2,702 (80.9)	1,575 (58.3)	1,127 (41.7)	3.17 (1.56, 6.46)^*^
Less than once a week	334 (10.0)	149 (44.6)	185 (55.4)	1.99 (0.90, 4.41)^*^
At least once a week	304 (9.1)	80 (26.4)	224 (64.6)	Ref.
**Reading newspaper**				
Not at all	3,100 (92.8)	1,753 (56.5)	1,347 (44.5)	5.38 (2.36,11.22)^*^
Less than once a week	167 (5.0)	37 (22.3)	130 (77.7)	1.15 (0.27, 4.99)
At least once a week	73 (2.2)	14 (19.4)	59 (80.6)	Ref.
**Own mobile phone**				
Yes	583 (17.5)	227 (38.9)	356 (61.1)	Ref.
No	2,757 (82.5)	1,577 (57.2)	1,180 (42.8)	2.67 (1.22, 5.83)^*^
**Covered by health insurance**				
Yes	139 (4.2)	45 (32.6)	94 (67.4)	Ref.
No	3,200 (95.8)	1,759 (55.0)	1,442 (45.0)	1.77 (1.16, 2.70)^*^
**Autonomy in decision-making**				
Low	548 (16.4)	307 (56.0)	241 (44.0)	0.96 (0.61, 1.51)
Moderate	388 (11.6)	212 (54.6)	176 (45.4)	1.04 (0.76, 1.41)
High	2,403 (72.0)	1,285 (53.4)	1,119 (46.6)	Ref.
**Ease of distance to seek medical care**				
Big problem	1,959 (58.7)	1,202 (61.4)	757 (39.6)	1.76 (1.25, 2.48)^*^
Not a big problem	1,381 (41.3)	601 (43.6)	779 (56.4)	Ref.
**Access to money for seeking medical care**				
Big problem	2,048 (61.3)	1,223 (59.7)	825 (40.3)	1.74 (1.27, 2.39)^*^
Not a big problem	1,292 (38.7)	581 (45.0)	711 (55.0)	Ref.

Ref, Reference category;

* significant at p < 0.25.

### The level of incomplete PCV across the regions

**Figure 1:**
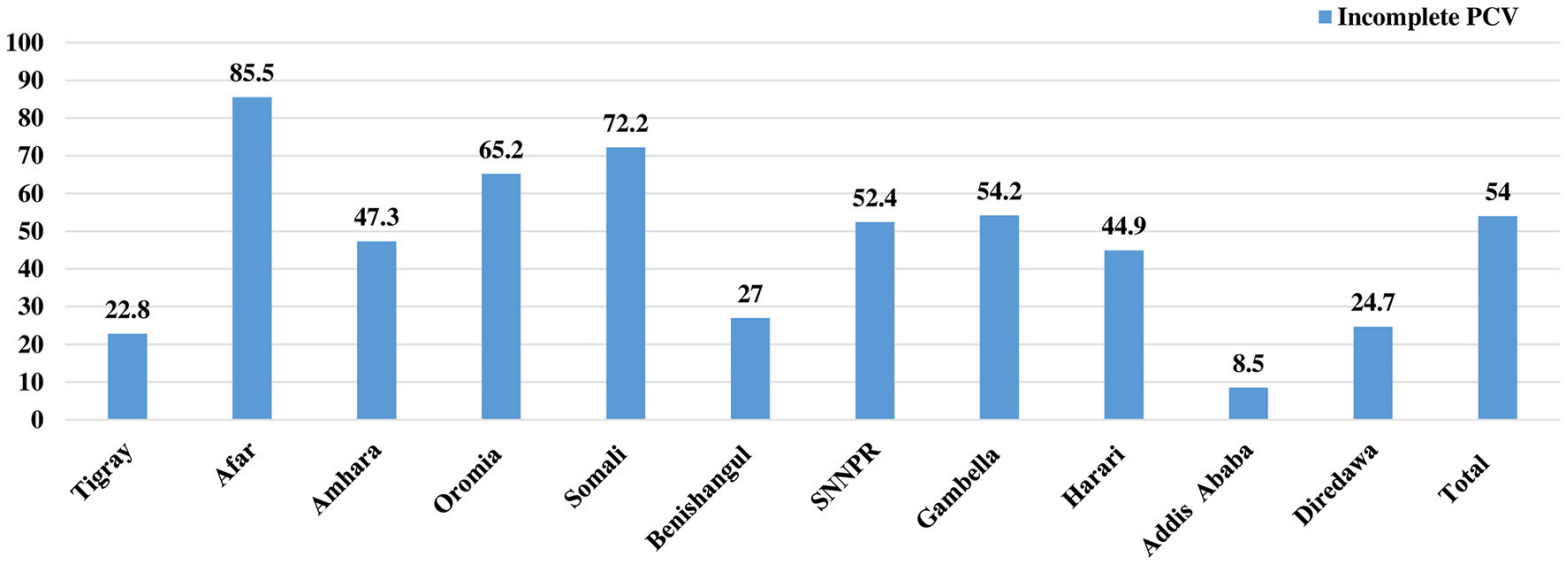
Disparities in the level of incomplete PCV across regions of Ethiopia, EDHS 2016.

Regarding the vaccination status, more than half, 54.0% (95% CI: 52.31, 55.69) of children were not fully vaccinated for the Pneumococcal conjugate vaccine (PCV). Afar region accounted for the highest proportion of children who were not fully vaccinated for PCV (84.5%), followed by Somali (72.2%) and Oromia (65.2%) regions. Addis Ababa, Tigray, and Diredawa regions, on the other hand, had the greatest proportions of children who were fully vaccinated for PCV, at 91.5%, 77.2%, and 75.3%, respectively (Figure 1).

### Spatial analysis results

#### Spatial distribution of incomplete PCV uptake

**Figure 2:**
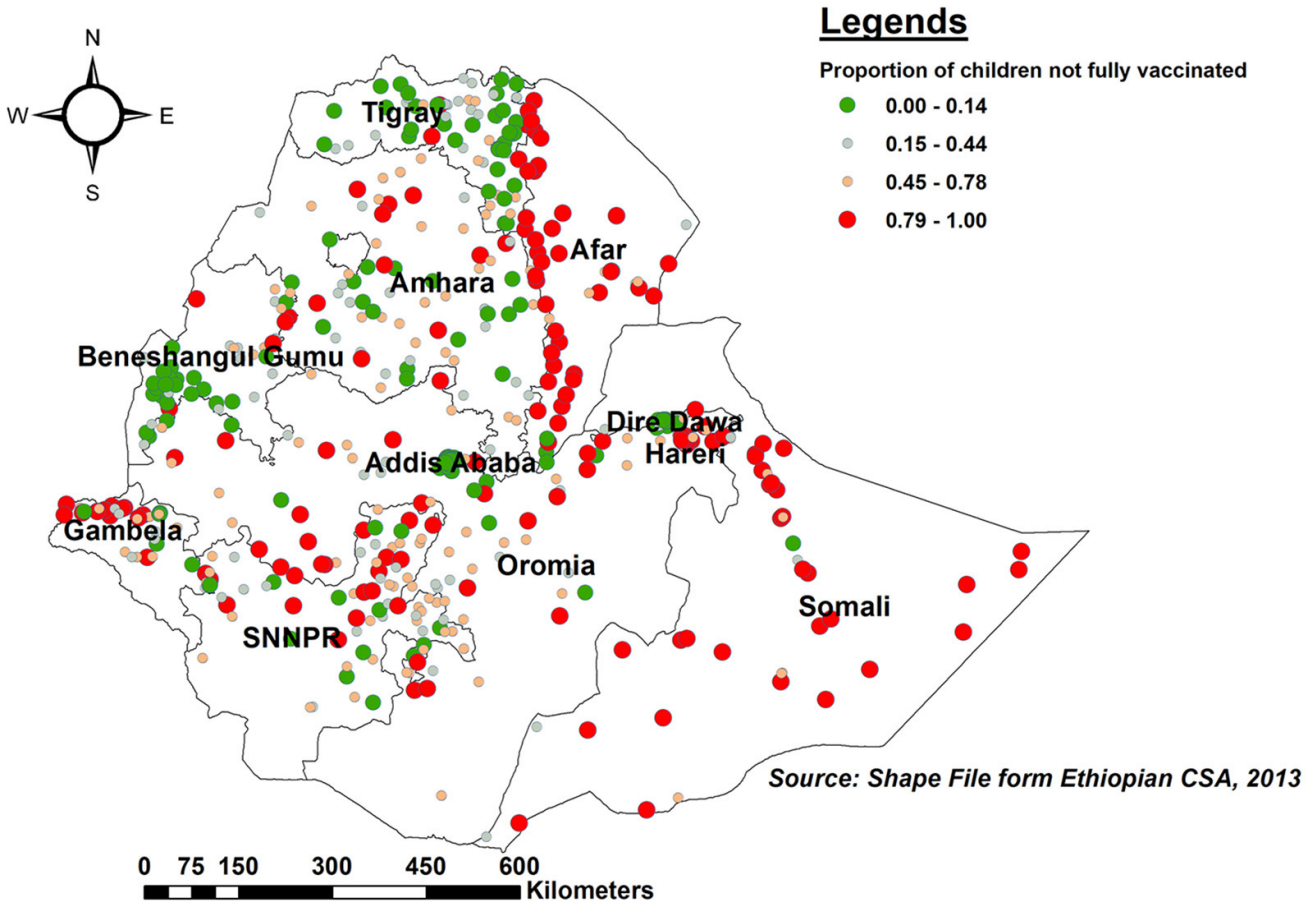
Spatial distribution of incomplete PCV uptake among children aged 12–35 months in Ethiopia, EDHS 2016.

The proportion of children with incomplete PCV uptake was higher in Somalia, the western border of Afar, northern and southern Amhara, northern Gambella, and SNNPR. In contrast, Tigray, Benishangul, central Addis Ababa, and the eastern border of Amhara had a lower proportion of children with incomplete PCV (Figure 2).

#### Spatial and incremental autocorrelation

**Figure 3:**
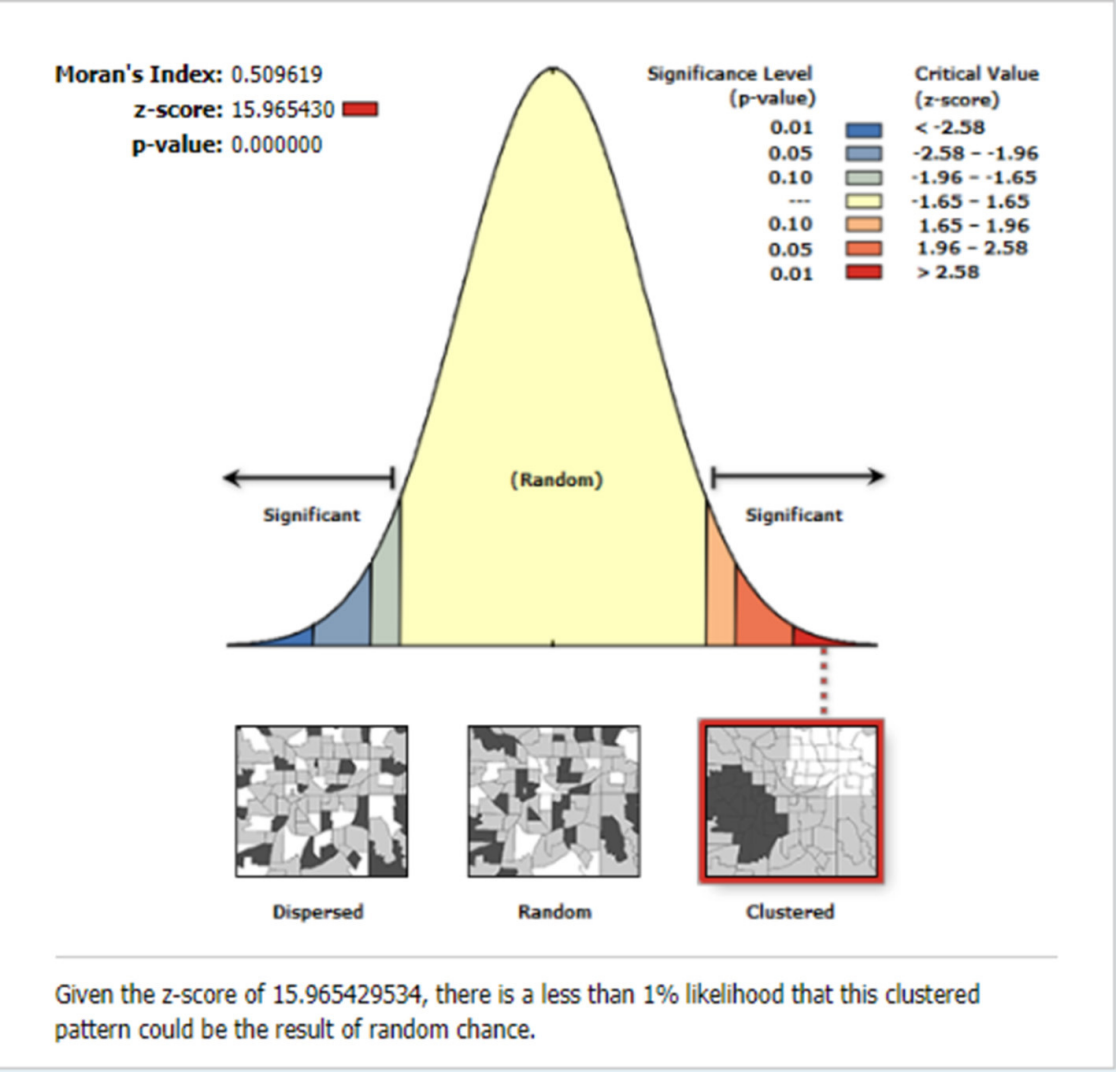
The global spatial autocorrelation of incomplete PCV uptake among children aged 12–35 months in Ethiopia, EDHS 2016.

**Figure 4:**
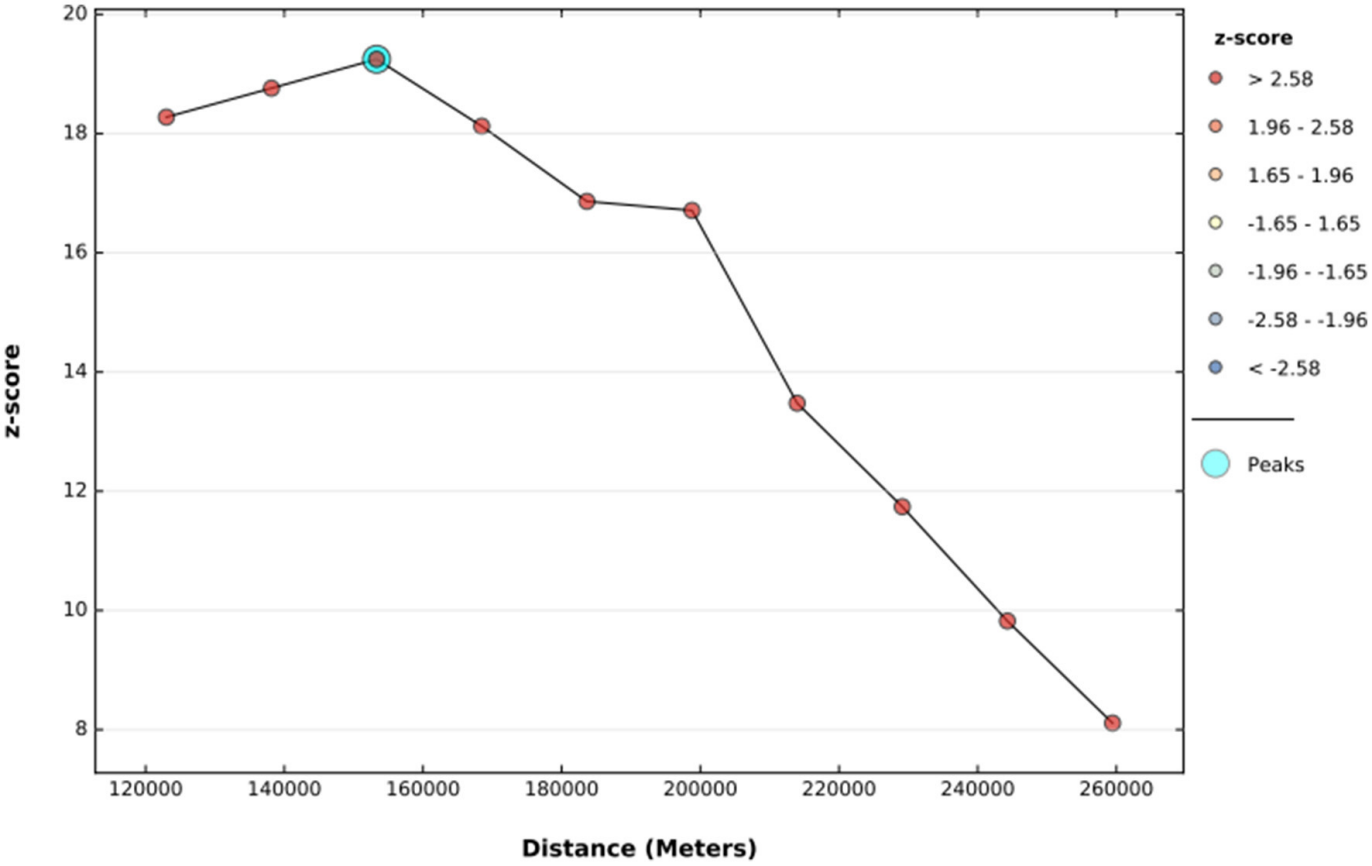
The incremental autocorrelation of incomplete PCV uptake among children aged 12–35 months in Ethiopia, EDHS 2016.

The global spatial autocorrelation result revealed that incomplete PCV uptake was non-random Ethiopia (Global Moran's I = 0.509, *p* < 0.001) (Figure 3). The incremental spatial autocorrelation across a series of distances was displayed by a line graph with a corresponding z-score to establish the average nearest neighbor and minimum and maximum distance band. A total of 10 distance bands were uncovered with an initial distance of 122,990.0 meters, with the first maximum peak (clustering) identified at 153,314.99 meters (Figure 4).

### Hot spot and cold spot (Getis-Ord Gi^*^) analysis

**Figure 5:**
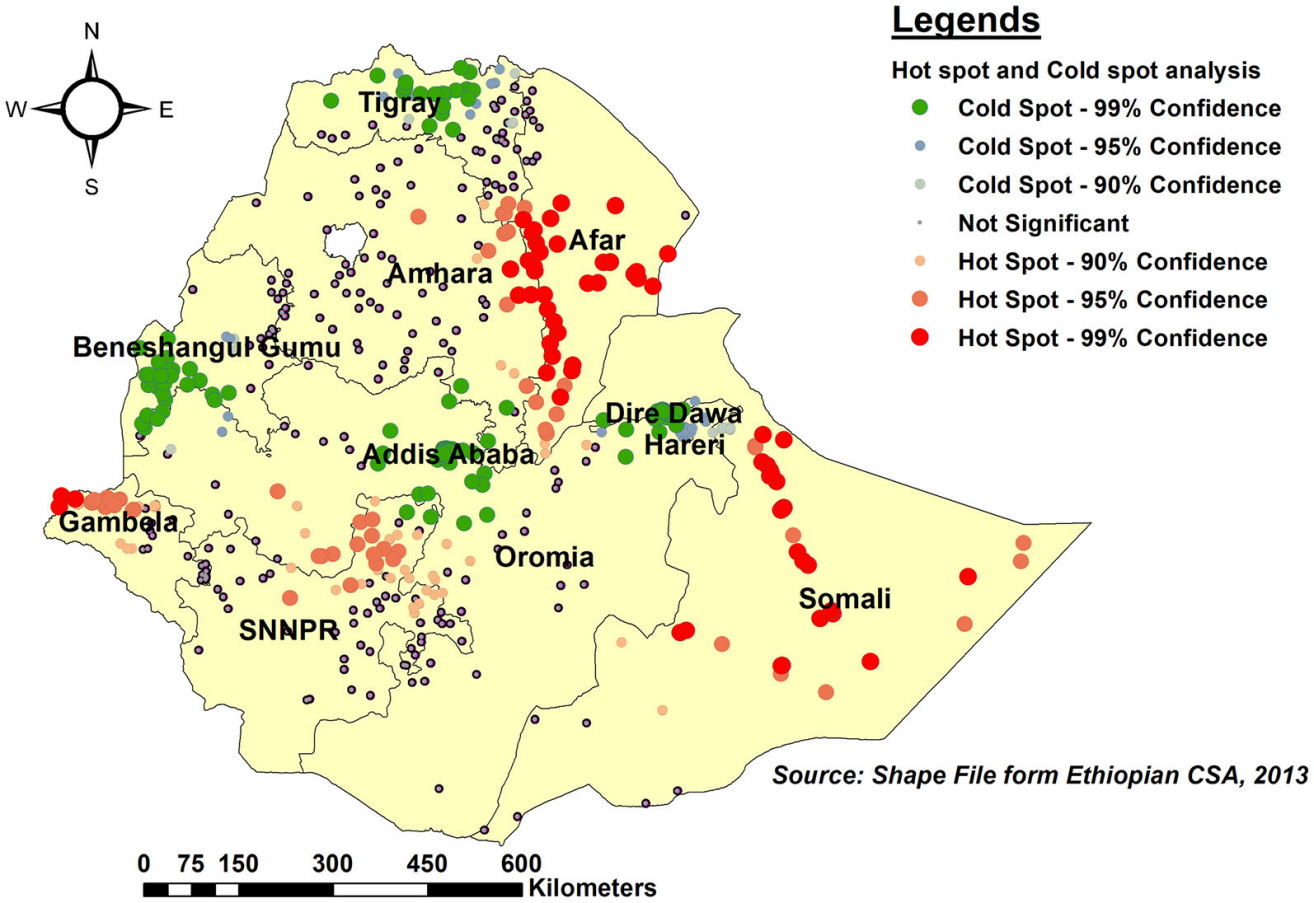
Hot spot and Cold spot analysis of incomplete PCV uptake among children aged 12–35 months in Ethiopia, EDHS 2016.

As per hot spot and cold spot analysis, the vast majority of Somali, southeast Afar, and eastern Gambela regions were hot spots (areas with a higher proportion of children with an incomplete PCV schedule), as indicated by red-colored points. Central Tigray, southwest Benishangul-Gumuz, Central Addis Ababa, and Dire Dawa, on the other hand, were cold spot areas (areas with lower rates of incomplete PCV uptake) (Figure 5).

### Spatial interpolation

**Figure 6:**
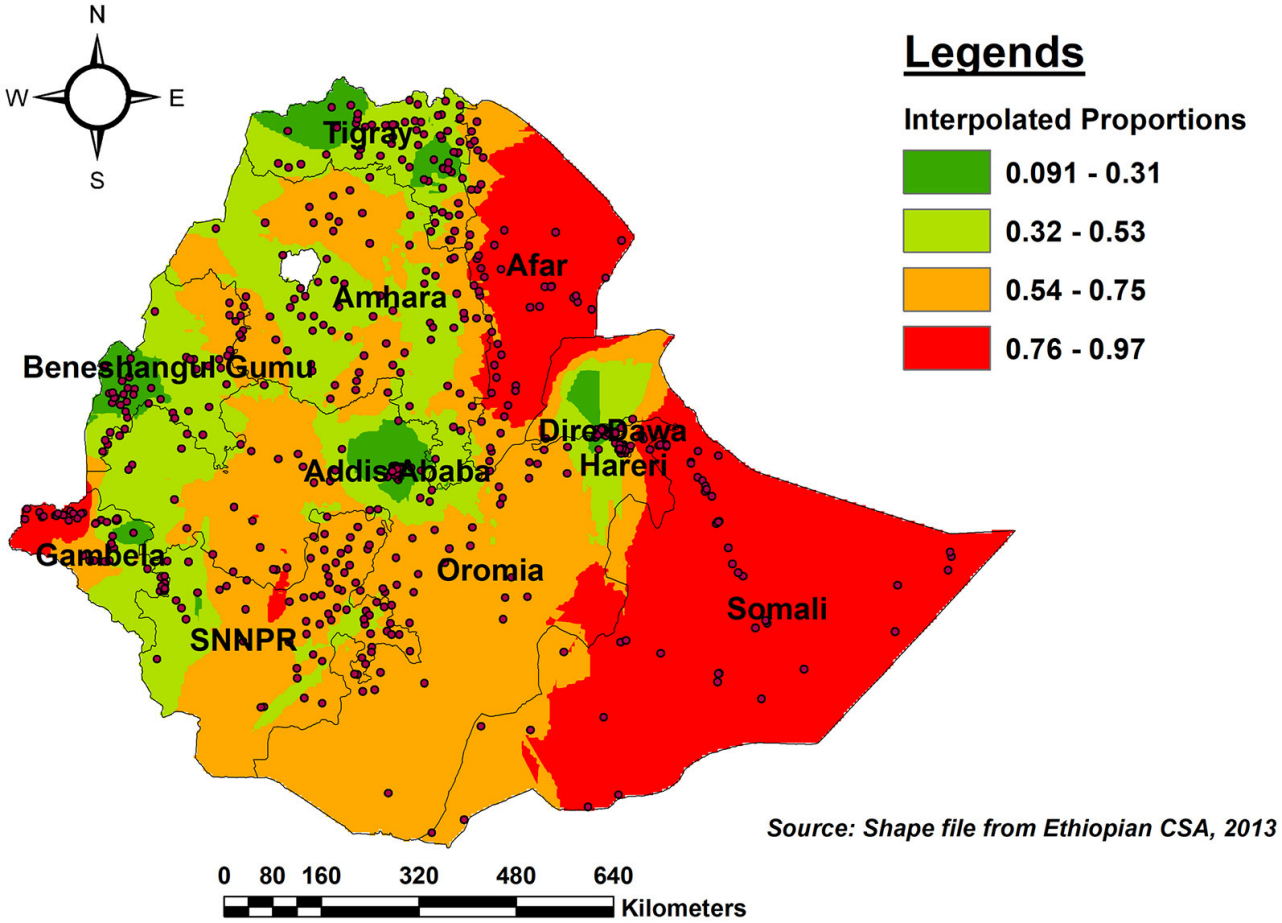
Ordinary Kriging interpolation of the spatial distribution of incomplete PCV uptake among children aged 12–35 months in Ethiopia, EDHS 2016.

As per the kriging interpolation results, the entire Somali region, the vast majority of Afar, and the western border of Gambella had a higher predicted proportion of incomplete PCV uptake. However, the predicted proportion of incomplete PCV uptake was lower in eastern and western Tgiray, Central Addis Abeba, southwest of Benishangul-Gumuz, and the northern part of Dire Dawa (Figure 6).

### Spatial scan statistical (SaTScan) analysis

The SaTScan spatial analysis detected a total of nine statistically significant SaTScan cluster areas with a high proportion of children who were not fully vaccinated for PCV, which means that the prevalence of incomplete vaccination for PCV is higher inside the SaTScan circular window compared to outside the SaTScan window. The most likely primary SaTScan cluster of areas with a higher rate of incomplete PCV was detected in the Somali region at geographical coordinates of (6.023458 N, 44.807507 E) with (LLR = 33.79, RR=1.76, *p* < 0.01). This revealed that children within the spatial window had 1.76 times higher odds of receiving incomplete PCV as compared to those children outside the spatial window (Supplementary File).

### Results of multilevel mixed effect multivariable logistic regression

#### Measures of variation (random effects) and model fit statistics

The ICC in the null model revealed that 42.2% of the variability in incomplete PCV uptake was explained by cluster variations (ICC = 0.422, *p* < 0.001). In addition, variation at the individual and community levels accounted for 32.7% (ICC = 0.0.327, *p* < 0.001) and 39.0% (ICC = 0.390, *p* < 0.001) of the variation in the probabilities of having incomplete PCV, respectively. The median odds ratio (MOR) indicates the amount to which the risk of having incomplete PCV is determined by clusters and is hence suitable for assessing contextual factors. In the current study, a MOR of 4.43 in the null model revealed that if we randomly selected a child from two different clusters, the child from the cluster with a higher rate of incomplete PCV uptake had 4.43 times higher odds of not being fully vaccinated as compared to the child from the cluster with a lower uptake. Furthermore, a proportionate change in variance (PCV) of 55.5% suggests that individual and community-level factors together accounted for 55.5% of the variability seen in the null model. As we moved from model 1 (the empty model) to model 4 (the full model), the values of AIC, BIC, and Deviance declined, indicating that the final model fitted throughout the study had appropriate goodness of fit. Finally, the fourth model with the lowest deviation (3,597.2) was selected as the best model fit for the study (Table 4).

**Table 4:** Measures of variation (random intercept models) and model fit statistics of incomplete PCV uptake among children aged 12–35 months in Ethiopia, EDHS 2016.

**Variable categories**	**Model I (null model)**	**Model II (individual-level factors)**	**Model III (community-level factors)**	**Model-IV (full model)**
**Random effects**				
Variance	2.43	1.60	2.18	1.09
ICC	0.424	0.327	0.390	0.248
AIC	3,973.8	3,668.2	3,892.8	3,459.2
BIC	3,985.9	3,838.5	3,923.2	3,647.8
MOR	4.43	3.34	4.1	2.34
PCV	Ref.	34.1%	10.2%	55.1%
**Model fitness**				
Log-likelihood	−1,984.9	−1,806.1	−1,941.3	−1,798.6
Deviance	3,969.8	3,612.2	3,882.6	3,597.2

#### Fixed effect (predictors of incomplete PCV uptake)

In the mixed-effect multilevel multivariable logistic regression analysis, living in peripheral regions, religion, not having ANC visits, home birth, never listening to the radio, and not owning a mobile phone were found to be significantly associated with incomplete PCV uptake. As compared to children from metropolitan regions, the odds of incomplete PCV uptake were 4.63 (AOR= 4.63; 95% CI: 2.34, 9.15) and 3.16 (AOR= 3.16; 95% CI: 2.06, 7.38) times higher for children from peripheral and major central regions, respectively. Children from Traditional and Muslim religious families had a 7.41(AOR = 7.41, 95% CI: 2.8, 13.18) and 2.62 (AOR = 2.62, 95% CI: 1.96, 3.51) times higher likelihood of not being fully vaccinated than children from Orthodox families, respectively. Children from mothers who did not receive ANC during their last pregnancy were 2.76 times more likely not to be fully vaccinated for PCV than children from mothers who received four or more ANC visits (AOR = 2.76, 95% CI: 1.91, 4.00). Children born to mothers who did not receive the Tetanus injection before childbirth were 1.84 times more likely to have an incomplete PCV schedule than children born to mothers who received at least a single shot (AOR = 1.84, 95% CI: 1.29, 2.74). Similarly, the odds of incomplete PCV vaccinations were 1.72 times higher among children delivered at home vs. their counterparts delivered in health facilities (AOR = 1.72, 95% CI: 1.23, 2.34). Children born to mothers who do not own a mobile phone were 1.64 (AOR = 1.64, 95% CI: 1.38, 1.93) times more likely to have incomplete PCV than their counterparts (Table 5).

**Table 5:** Results of a multilevel mixed-effect multivariable logistic regression to identify the determinants of failure to take and/or missing PCV in Ethiopia, EDHS 2016.

**Variable categories**	**Model I (null model)**	**Model II (individual-level factors)**	**Model III (community-level factors)**	**Model-IV** **(full model)**
		IRR (95%CI)	IRR (95%CI)	IRR (95%CI)
**Religion**				
Muslim		2.90 (1.95, 4.32)^*^		2.62 (1.96, 3.51)^*^
Protestant		1.36 (0.90, 2.05)		1.23 (0.92, 1.66)
Catholic		1.89 (0.58, 6.09)		1.65 (0.66, 4.17)
Others/traditional		8.69 (2.25, 23.45)^*^		7.41 (2.8, 13.18)^*^
Orthodox		Ref.		Ref.
**Wealth index**				
Poorest		1.55 (0.77, 3.12)		1.43 (0.68, 2.99)
Poorer		1.47 (0.73, 2.95)		1.36 (0.65, 2.83)
Middle		1.76 (0.88, 3.54)		1.62 (0.77, 3.39)
Richer		1.52 (0.82, 2.79)		1.38 (0.72, 2.64)
Richest		Ref.		Ref.
**Educational status**				
No education		1.86 (0.84, 4.13)		1.81 (0.79, 4.12)
Primary		1.44 (0.70, 2.97)		1.41 (0.66, 2.98)
Secondary and higher		Ref.		Ref.
**Parity**				
Primiparous		Ref.		Ref.
Multiparous		0.66 (0.43, 1.03)		0.66 (0.42, 1.03)
Grand multiparous		0.83 (0.53, 1.30)		0.83 (0.53, 1.30)
**Frequency of ANC**				
No visit		2.80 (1.94, 4.06)^*^		2.76 (1.91, 4.00)^*^
One visit		2.09 (1.33, 5.83)^*^		1.86 (1.21, 4.80)^*^
2–3 visits		1.23 (0.85, 1.79)		1.20 (0.82, 1.74)
≥4 visits		Ref.		Ref.
Tetanus vaccine uptake				
Yes		Ref.		Ref.
No		1.88 (1.29, 2.74)		1.84 (1.29, 2.74)^*^
**Place of delivery**				
Home		2.59 (1.37, 3.36)^*^		1.72 (1.23, 2.34)^*^
Health facilities		Ref.		Ref.
Postnatal care service				
Yes		1.24 (0.71, 1.96)		1.14 (0.81, 1.76)
No		Ref.		Ref.
**Listen to radio**				
Not at all		1.67 (1.13, 2.73)^*^		1.67 (0.92, 2.65)
Less than once a week		1.10 (0.81, 1.74)		1.14 (0.65, 1.98)
At least once a week		Ref.		Ref.
**Watching TV**				
Not at all		0.75 (0.31, 1.80)		0.83 (0.50, 1.38)
Less than once a week		0.98 (0.40, 2.36)		0.97 (0.58, 1.63)
At least once a week		Ref.		Ref.
**Reading newspaper**				
Not at all		1.47 (0.68, 5.06)		1.63 (0.65, 4.03)
Less than once a week		0.93 (0.39, 2.19)		0.87 (0.38, 2.22)
At least once a week		Ref.		Ref.
**Own mobile phone**				
Yes		Ref.		Ref.
No		1.67 (1.39, 2.15)		1.64 (1.38, 1.93)^*^
**Covered by health insurance**				
Yes		Ref.		Ref.
No		1.98 (0.83, 4.72)		2.03 (0.85, 4.81)
**Ease of distance to seek medical care**				
Big problem		1.25 (0.86, 1.81)		1.24 (0.85, 1.80)
Not a big problem		Ref.		Ref.
**Access to money for seeking medical care**				
Big problem		1.31 (0.90, 1.91)		1.33 (0.91, 1.94)
Not a big problem		Ref.		Ref.
**Regions**				
Peripheral			11.08 (5.75, 21.33)^*^	4.63 (2.34, 9.15)^*^
Major central regions			3.93 (2.09, 7.40)^*^	3.16 (2.06, 7.38)^*^
Metropolitans			Ref.	Ref.
**Residence**				
Rural			3.57 (2.05, 6.20)^*^	0.90 (0.42, 1.90)
Urban			Ref.	Ref.

Ref., Reference category; AOR, Adjusted Odds Ratio;

* statistically significant at p < 0.05.

## Discussion

In this study, the proportion of children in Ethiopia who did not receive the complete schedule of Pneumococcal conjugate vaccination (PCV) was found to be 54.0% (95% CI: 52.31, 55.69), which was higher than a similar study conducted in Ethiopia among children aged 12–23 months (50.9%) (17). This result lags far behind the EPI and the Global Vaccine Action Plan (GVAP), which set national vaccine coverage goals of 90% by 2020 (11). Global spatial Moran's investigation found that the proportion of children who missed the full PCV schedule varied significantly across regions d (Global Moran's I = 0.509, *p* < 0.0001). As per hot spot analysis, statistically significant hotspot areas for missing complete PCV were the vast majority of Somali, southeast Afar, and eastern Gambela regions. These regions were known for having a low coverage of routine vaccination (17, 35–37). This could be owing to geographical and logistics challenges, as these regions are known for their harsh climate and limited healthcare infrastructures, making it difficult to reach remote areas with vaccination programs, which could impede vaccine delivery (38, 39). Furthermore, due to a shortage of healthcare providers, access to up-to-date information about vaccines and their advantages may be limited, potentially leading to noncompliance with recommended immunization schedules. This high level of incomplete childhood PCV coverage may indicate that not only the country but also regions with high levels of childhood PCV coverage are nonetheless at high risk for pneumococcal infections in children due to low herd immunity (40).

According to the multilevel analysis, the likelihood of missing the full PCV schedule was attributable to both individual and community-level characteristics. Accordingly, living in peripheral regions, not having ANC visits, not having pre-birth Tetanus injection, home birth, and not owning a mobile phone were found to be significantly associated with incomplete PCV uptake.

Children from the peripheral regions (Afar, Somali, and Gambella) had a higher chance of not being fully vaccinated for PCV. This was supported by studies conducted in Ethiopia (7, 35, 41). This could be owing to difficulty in accessing health services, inadequate knowledge of childhood immunization, socio-cultural, and logistical difficulties, as well as differences in socio-cultural and religious backgrounds (41). Furthermore, because the majority of people in these regions live pastoral or partially nomadic lives (39), women may find it difficult to adhere to recommended vaccination schedules as their places of residency shift over time. As a result, the federal and regional governments should work together to build mobile vaccination, plan vaccination campaigns, and work with local organizations and non-governmental organizations to minimize incomplete PCV uptake.

Failure to take complete PCV was associated with a lack of maternal health services, like ANC, skilled delivery service, and pre-birth tetanus injection. Lack of ANC was found to be a significant predictor of an incomplete PCV schedule. This was supported by studies conducted in Afghanistan (42), Indonesia (43), Nigeria (44), Malawi (45), Benin (46), and Ethiopia (17, 47, 48). ANC is a crucial entrance point into the healthcare system for pregnant women and their families to receive a broad range of health promotion and prevention services (49). ANC allows healthcare providers to educate expecting mothers about the importance, timing, and place of childhood vaccination. Without ANC, women may not have this essential information, leading to noncompliance with routine child health services like immunization. The positive relationship between no ANC and incomplete PCV coverage highlights the need for Ethiopia's Ministry of Health to improve ANC provision for pregnant women. Likewise failure to receive a pre-birth tetanus injection was also associated with incomplete vaccination, which was supported by studies conducted in Myanmar (50), Indonesia (51), and Ethiopia (52). Similarly home birth also significantly contributes to incomplete PCV uptake. Studies conducted in Indonesia (43), Myanmar (50), Pakistan, Benin (46), Senegal (53), and Ethiopia (48) were in tandem with the current finding. This could be owing to limited access to healthcare services, as the majority of women who give birth at home may come from remote areas to health facilities, resulting in lower vaccination rates. Furthermore, when women give birth outside of healthcare institutions, they frequently do not receive counseling or information on the need for child immunizations which could result in noncompliance. Thus, the Ethiopian Federal Ministry of Health needs to focus on improving the provision of maternal health services in order to enhance immunization coverage of newly introduced vaccines. This can be achieved through targeted outreach efforts, improving healthcare infrastructure, and increasing community engagement and awareness.

The odds of an incomplete PCV schedule were higher among children from mothers without mobile phones. Studies conducted in Bangladesh (54), and Ethiopia (55). Mobile phones contribute to vaccine compliance by delivering SMS reminders for vaccination appointments and offering information about vaccine schedules, the importance of vaccines, and their benefits (56). Community health workers or healthcare practitioners in Ethiopia continue to use mobile phones for exchanging health-related communications, especially vaccine-related information (57). As a result, women or caretakers without a mobile phone may miss timely reminders on when and where to take their child for vaccination, resulting in missed appointments.

The study has the following strengths. To begin, the findings relied on an analysis of nationally representative data with a high response rate, which made the findings more generalizable. The findings of Hot Spot, SaTScan, and multilevel mixed-effect logistic regression analysis can help government and program planners develop geographic, individual, and community-focused public health initiatives that tackle the level of incomplete PCV. However, the finding should be seen in light of its limitations. First of all, as EDHS data is cross-sectional, it is difficult to establish a temporal/causal relationship between the outcome and explanatory variables. In addition, since the data was collected through retrospective interviews of women, the findings may be exposed due to recall bias.

## Conclusion

The level of incomplete PCV schedule was found to be high in Ethiopia with a significant spatial variation across regions. The vast majority of Somali, southeast Afar, and eastern Gambella regions were statistically significant hot spots for incomplete PCV. Lacking ANC visits, not getting pre-birth Tetanus injections, home birth, not possessing a mobile phone, and residing in a peripheral region were identified as statistically significant predictors of incomplete PCV. Hence, the federal and regional governments should collaborate with NGOs to improve vaccination coverage and design strategies to trace those children with incomplete PCV in peripheral regions. In addition, it's imperative that policymakers and maternal-child health program planners implement together territorial strategies by enhancing access to maternal and child health services to increase in a few months of coverage vaccines.

## Data availability statement

The raw data supporting the conclusions of this article will be made available by the authors, without undue reservation.

## Ethics statement

The studies involving humans were approved by Wachemo University Ethics Committee, Department of Public Health. The studies were conducted in accordance with the local legislation and institutional requirements. Written informed consent for participation in this study was provided by the participants' legal guardians/next of kin.

## Author contributions

AH: Conceptualization, Formal analysis, Methodology, Project administration, Software, Validation, Visualization, Writing – original draft, Writing – review & editing. SH: Methodology, Software, Writing – review & editing. LT: Methodology, Software, Writing – review & editing. BW: Formal analysis, Methodology, Writing – review & editing.

## Supplementary material

Supplementary File The most likely SaTScan clusters of areas with a high prevalence of incomplete PCV uptake among children aged 12–35 months in Ethiopia, EDHS 2016.
