# Supplementary material for: Spatial variation and predictors of incomplete pneumococcal conjugate vaccine (PCV) uptake among children aged 12–35 months in Ethiopia: spatial and multilevel analyses
Source: Front Public Health. 2024 May 28;12:1344089. doi: 10.3389/fpubh.2024.1344089 (PMC11165216; doi:10.3389/fpubh.2024.1344089)
Supplement: Supplementary File — The most likely SaTScan clusters of areas with a high prevalence of incomplete PCV uptake among children aged 12–35 months in Ethiopia, EDHS 2016. [file Table_1.DOCX]

| Most likely clusters | Enumeration areas (clusters) identified | Number of clusters | Population | No. of  case | Coordinates / Radius | Relative risk | LLR | P-Value |
| --- | --- | --- | --- | --- | --- | --- | --- | --- |
| 1^st^ most likely cluster | 146, 138, 92, 490, 543, 492, 85, 358, 164, 77, 171, 198, 629, 95, 497, 278, 521, 588, 458, 553, 269, 318, 187, 630, 214, 251, 573, 556, 239, 116, 22, 520, 33, 568, 277, 480, 527, 208, 64, 439, 57, 8, 210, 186, 394, 454, 436, 566, 212, 501 | 50 | 275 | 231 | (6.023458 N, 44.807507 E) / 463.24 km | 1.76 | 72.45 | <0.001 |
| 2^nd^ most likely cluster | 4, 632, 75, 596, 440, 366, 178, 499, 205, 427, 334, 570, 348, 599, 544, 389, 368, 241, 55, 547, 191, 571, 344, 276, 332, 189, 254, 37, 249, 620, 488, 307, 135, 345, 283 | 35 | 197 | 172 | (11.845228 N, 41.915793 E) / 242.50 km | 1.8 | 62.7 | <0.001 |
| 3^rd^ most likely cluster | 104, 260, 592, 507, 233, 69, 370, 426, 603, 346, 315, 536, 435, 309, 567, 343, 266, 105, 618 | 19 | 114 | 95 | (8.309769 N, 33.805118 E) / 63.79 km | 1.68 | 27.3 | <0.001 |
| 4^th^ most likely cluster | 362, 127, 235, 263 | 4 | 35 | 35 | (13.889667 N, 39.944065 E) / 16.79 km | 1.99 | 23.87 | <0.001 |
| 5^th^ most likely cluster | 172, 130, 511, 421, 585 | 5 | 26 | 26 | (13.248133 N, 40.043685 E) / 44.40 km | 1.98 | 17.69 | <0.001 |
| 6^th^ most likely cluster | 432, 486, 62, 447, 227, 76, 489, 586 | 8 | 53 | 46 | (7.858150 N, 36.733552 E) / 78.67 km | 1.73 | 15.64 | <0.001 |
| 7^th^ most likely cluster | 515, 615 | 12 | 19 | 19 | (11.074357 N, 36.455218 E) / 8.94 km | 1.98 | 12.91 | 0.002 |
| 8^th^ most likely cluster | 506, 412, 476, 333, 491, 122, 372, 51, 49, 71, 93, 564, 230, 245, 529, 39, 336, 484, 441 | 19 | 119 | 87 | (9.120627 N, 40.753382 E) / 115.32 km | 1.46 | 12.77 | 0.0021 |
| 9^th^ most likely cluster | 327, 152, 312, 640, 163, 638, 628, 322, 199, 80, 512 | 11 | 65 | 50 | (9.120627 N, 40.753382 E) / 115.32 km | 1.53 | 9.55 | 0.041 |
